# Impact of Type 1 Diabetes on Endothelial Cells Derived From Living Donors

**DOI:** 10.1096/fba.2025-00104

**Published:** 2025-11-05

**Authors:** Isra Marei, Maria Vinokurova, Li Qiucheng, Lasse Nyhegn, Eric Dubuis, Anam Baig, Nura Mohamed, Stephen Rothery, Nicholas S. Kirkby, Victoria Salem, Nicholas Oliver, Jane A. Mitchell, Blerina Ahmetaj‐Shala

**Affiliations:** ^1^ National Heart and Lung Institute Imperial College London London UK; ^2^ Department of Pharmacology Weill Cornell Medicine–Qatar Doha Qatar; ^3^ Biomedical Research Centre Qatar University Doha Qatar; ^4^ Facility for Imaging by Light Microscopy Imperial College London London UK; ^5^ Department of Bioengineering Imperial College London London UK; ^6^ Department of Metabolism, Digestion and Reproduction Imperial College London London UK

**Keywords:** cardiovascular pathogenesis, endothelial cells, inflammation, regenerative medicine, type 1 diabetes, vascular leak

## Abstract

Endothelial colony forming cells (ECFCs) derived from peripheral blood have been shown to retain disease phenotype in several conditions thus possessing great translational potential for regenerative medicine. Hyperglycaemia may alter the phenotype of ECFCs yet the characteristics of ECFCs isolated from people with type 1 diabetes (T1D) have not been described. Here, we establish whether ECFCs can be successfully isolated from donors with T1D and we characterize their functional properties. Human ECFCs were isolated from peripheral blood of up to 9 control and T1D donors. Expression of cell markers and cytokines was analyzed using immunocytochemistry, RT‐PCR and ELISA. Ca^2+^ signaling and contraction were studied using Fluo‐4‐AM in cells treated with serial concentrations of histamine (1 × 10^−7^‐1 × 10^−4^M). T1D ECFCs showed robust endothelial marker expression and displayed normal morphology but had a reduced size compared to those from control donors. In response to inflammatory stimuli, T1D ECFCs exhibited exaggerated pro‐atherogenic/pro‐inflammatory cytokine (IL‐6 and MCP‐1) and adhesion molecule gene induction (VCAM‐1 and ICAM‐1) but suppressed induction of interferon signaling markers (IP‐10). Histamine stimulated a concentration‐dependent increase in Ca^2+^ influx in ECFCs which was significantly reduced in ECFCs from T1D donors, independent of differences in H1 receptor expression levels. Histamine‐induced contraction was significantly enhanced in T1D ECFCs. ECFCs from control and T1D donors exhibit distinct phenotypic differences redolent of the vascular pathologies associated with T1D. This establishes the utility of T1D ECFCs for modeling vascular complications but also highlights the need to understand the potential limitations of autologous ECFCs to treat diabetic complications.

## Graphical abstract

Endothelial colony‐forming cells (ECFCs) were cultured from healthy volunteers (HV) and people with type‐1 diabetes (T1D). T1D ECFCs expressed robust endothelial cell markers (PECAM, VWF and NOS3) and normal morphology, but had a reduced size compared to HV. T1D ECFCs showed an increased gene expression of pro‐inflammatory cytokines (IL‐6, MCP‐1) and adhesion molecules (VCAM‐1, ICAM‐1) but suppressed induction of IP‐10. In response to histamine T1D ECFCs showed a reduced uptake of calcium, and histamine‐induced contraction was increased compared to HV.

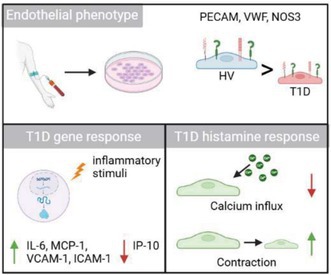

## 1 Introduction

Endothelial cells that line the inside of blood vessels form the main physical barrier between blood components and surrounding tissues. They play a major role in cardiovascular homeostasis by regulating vascular tone, permeability and immune responses [1]. Diabetes mellitus, a metabolic disease characterized by hyperglycaemia, is well known to be associated with widespread endothelial dysfunction and vascular complications, although the various mechanisms for this remain to be fully elucidated [2]. Diabetes affects 537 million (1 in 10) adults globally [3]: the major drivers of excess morbidity and mortality in diabetes are micro‐ and macrovascular complications. About 90% of people living with diabetes have type 2 diabetes (T2D), a condition driven by resistance to the action of insulin such that the ensuing hyperglycaemia is also associated with a range of dysmetabolic and inflammatory sequelae that converge to cause a 2–3‐fold increased risk of cardiovascular complications [4]. In contrast, type 1 diabetes (T1D) typically presents early in life, resulting from the autoimmune destruction of pancreatic beta cells and absolute deficiency of endogenous insulin production. People with T1D tend not to suffer the same levels of obesity, dyslipidaemia and insulin resistance as seen in T2D, yet they do develop a great burden of particularly microvascular disease. In fact, over 35% of adolescents diagnosed with T1D already exhibit some form of endothelial dysfunction [5].

Whilst hyperglycaemia per se is shared between different types of diabetes, a huge array of other pathophysiological mechanisms differ. T1D affects over 20 million people globally and its incidence is growing [2]. Other than trying to achieve very tight glycaemic control, which is associated with a huge mental load and also the risk of hypoglycaemia, there are remarkably few treatments directed at reducing microvascular damage in T1D. Moreover, as these patients age, it is still not fully understood how best to intervene to reduce the increasing risk of macrovascular disease [6]. Recently there has been a huge drive toward cell‐based therapies for T1D, with a particular focus on stem‐cell‐derived beta cell replacement [7]. One of the major barriers to the successful translation of this therapy is the failure of the cells to implant and vascularise, a problem mirrored with low success rates observed in cadaveric islet transplant [8]. Major efforts are now ongoing to attempt to augment islet implantation with the co‐transplant autologous endothelial or stromal support cells [9]. Therefore, both in terms of developing better treatments to target complications or advanced therapies for beta‐cell replacement, the study of endothelial cells in T1D is of central importance, and relatively understudied because of the difficulty in obtaining this tissue non‐invasively from donors with T1D.

Endothelial colony forming cells (ECFCs; also known as ‘blood outgrowth endothelial cells’ or BOECs) grown from endothelial progenitors in the blood provide an opportunity to liquid biopsy endothelium of living donors [10]. Over two decades since their initial discovery, ECFCs have been shown to have a robust phenotype compared to endothelial cells obtained directly from blood vessel walls [11] making them widely accepted in tissue engineering [12, 13] and gene delivery [14] and, as we have shown, are applicable for autologous cell platforms [15]. ECFCs have also been posited as advanced therapeutic options for the vascular complications of T1D [16, 17]. Another key advantage of ECFCs is that they can be used to phenotype the effect of disease in living donors on endothelial function. In this regard, ECFCs have been studied from patients with cardiovascular diseases including coronary artery disease, hereditary haemorrhagic telangiectasia, congenital bicuspid aortic valve, pulmonary arterial hypertension and venous thromboembolism [18]. Critically in many of these conditions, ECFCs cultured in vitro appear to retain disease phenotypes of the donor making them a unique platform to understand and model associated vascular dysfunction. However, research using ECFCs in the field of diabetes has been limited. Studies which have isolated ECFCs from people with gestational diabetes demonstrate reduced tubulogenic capacity, reduced migration toward chemotactic factors and reduced proliferation [19, 20]. ECFCs isolated from donors with T2D display improved in vitro proliferation and migratory capacity when pre‐treated with adiponectin [21]. However, to date there is no publication focused on the characterization of ECFCs from donors with T1D. It remains unknown what effects T1D has on basic morphological characteristics and other ‘responsive’ endothelial cell functions. In order to understand whether T1D ECFCs are a useful model for studying vascular complications, we also studied their responses to inflammatory stimuli. Inflammatory markers including C‐reactive protein (CRP), Interleukin (IL)‐6, IL‐8 and Tumor Necrosis Factor (TNF)‐α are strongly associated with microvascular complications in T1D [22] and inflammatory endothelial dysfunction is increasingly recognized as an important manifestation of T1D [23]. Histamine, released by mast cells and basophils, is a biologically active mediator known to affect endothelial cell immune response and barrier function. In diabetes, the well‐known vasoactive properties and effects on permeability leakage of histamine have been correlated with microvascular complications [24]. In humans, plasma and leukocyte histamine content is elevated in diabetes (including T1D) and is claimed to contribute to the underlying pathogenesis evoking endothelial permeability. These findings are in keeping with in vivo studies of experimental diabetes in rats suggesting an increased histaminergic tone and increased histamine content at key anatomical regions involved in long‐term diabetic complications, including the kidney, brain, lung, heart, pancreas and intestine [25].

Here, we have used ECFCs obtained from people with T1D to specifically address the effect of disease on phenotypic parameters, including the expression of key adhesion molecules and cytokines/chemokines, calcium (Ca^2+^) signaling and contraction in response to inflammation. This is of importance given the particular nature of vascular dysfunction in T1D and the need to develop these cells for regenerative applications in this condition.

## 2 Materials and Methods

### 2.1 Cell Culture

Human ECFCs were grown from peripheral blood mononuclear cells (PBMCs) isolated from up to 50 mL of blood collected from up to 9 healthy volunteers (5 males/4 females; aged 31.4 ± 1.4 years) and 9 donors with T1D (5 Males/4 Females; aged 46.2 ± 7.0 years) (full demographics shown in Table 1). PBMCs isolated from whole blood collected in BD Cell Preparation Tubes or BD Vacutainer tubes containing sodium heparin/Ficoll (BD Biosciences, UK) were plated and maintained in endothelial cell growth media‐2 (EGM‐2; Lonza, Germany: glucose concentration 5.5 mM) supplemented with 10% FBS (LabTech, UK) as previously described [26]. Once colonies had emerged, the FBS concentration in EGM‐2 was reduced to 2% and, unless otherwise stated, cells were maintained in this media.

**TABLE 1:** Demographics of donors that yielded ECFCs. ECFCs were isolated from control donors and donors with T1D for further investigation. Donor characteristics for both groups are highlighted. Values shown are ± SEM.

	T1D	HV
Number (*n*)	9	9
Age (years)	46.2 ± 7.0	31.4 ± 1.4
Gender	5 M; 4F	5 M: 4F
*For T1D only*		
Duration living with diabetes (years)	27.1 ± 6.7	
HbAc1 (mmol/mol)	69.4 ± 8.0	
Glucose (mmol/L)	7.5 ± 1.0	
Statin therapy	Yes (78%; 7/9)	
ACE inhibitors	Yes (33%; 3/9)	
Retinopathy	Yes (22%; 2/9)	

### 2.2 Study Approval

Ethical approval was obtained from (i) Imperial College Research Ethics Committee (19IC5372) and (ii) REC Wales approval (17/WA/0161) as per the Declaration of Helsinki. Informed written consent was given prior to the inclusion of subjects in the study. All cells were used between passages 3 and 9. Individual numbers for donors used in experiments are described in figure legends.

### 2.3 Immunostaining

ECFCs plated (50,000 cells/well) onto 24‐well polymer glass‐like bottom plates (Ibidi, Germany) were fixed with 4% paraformaldehyde (Sigma‐Aldrich, UK) for 15 min and permeabilised with 0.1% triton (Merck, Germany) for 5 min. Cells were blocked with 3% bovine serum albumin (BSA; Sigma‐Aldrich, UK) for 30 min. For cell staining, all the antibodies/dyes were diluted in PBS containing 1% BSA and incubated for 1 h in the dark. A description of the antibodies used, and specific dilutions is shown in Table S1. Stained cells were imaged (3–4 images/well) using a Zeiss Axio Observer microscope (Carl Zeiss, Germany) and a 20 × 0.8na objective with an appropriate LED on a Hamamatsu Flash 4 camera. All fluorescence settings were kept the same throughout and the intensity of images quantitatively analyzed using a bespoke macro‐script (https://www.imperial.ac.uk/medicine/facility‐for‐imaging‐by‐light‐microscopy/software/fiji/) for Fiji software (v2.9) which, for each image, calculated the ‘total intensity’ by measuring the overall light intensity and subtracting the background before normalizing by the number of cells (DAPI counted).

### 2.4 ECFC Inflammatory Stimulation

ECFCs were plated (2 × 10^5^ cells/well) in a 6‐well plate in EGM2/2% FBS overnight. The next day the media was replaced with new media containing either 10 ng/mL TNF‐α (Thermo Fisher, UK), 10 ng/mL IL‐1ß (R&D Systems, UK), 10 μg/mL Pam3CSK4 (Tocris, UK), 1 μg/mL LPS (Sigma‐Aldrich, UK), or vehicle (EGM2/2% FBS) for 24 h. After this, cell culture supernatant was removed and RNA was extracted for respective ELISA and real‐time quantitative polymerase chain reaction (RT‐qPCR).

### 2.5 Rt‐PCR

mRNA was collected using the RNeasy Mini Kit (QIAGEN, Germany). Gene expression was measured using a one‐step RT‐qPCR mix (Promega, UK) and TaqMan gene expression probes for CD31/PECAM1, VWF, NOS3, IL6, CXCL10, CCL2, ICAM1, VCAM1, SELP, HRH1, PCNA, ENG, MK167, GAPDH and 18S (Thermo Fisher, UK) in 96‐well reaction plates (Thermo Fisher, UK). RT‐PCR was carried out using the Thermo Fisher (UK) StepOnePlus RT PCR System. For data analysis, cycle threshold (CT) values were processed by the 2^−∆∆Ct^ method using an average housekeeper correction for 18S and GAPDH. Raw Ct values per sample are shown in Table S2.

### 2.6 Ca^2+^ Signaling and Data Analysis

ECFCs plated (15,000 cells/well) in 24‐well polymer glass‐like clear bottom black plates (Ibidi, Germany) were loaded with Fluo‐4AM (1.1 × 10^−6^ M) containing non‐ionic polyols surfactant (Powerload, Life Technologies, UK) in extra‐cellular solution (ECS; 1.36 × 10^−1^ M NaCl, 5.4 × 10^−3^ M KCl, 1 × 10^−3^ M MgCl_2_, 3.3 × 10^−4^ mM NaH_2_PO_4_, 1 × 10^−2^ M HEPES, 1 × 10^−2^ M D‐glucose, and 2.5 × 10^−2^ M CaCl_2_) for 40 min at 37°C in the dark. Next, cells were washed twice with PBS and returned to a dark incubator for 30 min to stabilize in ECS. Cells were then treated with ECS only (control), U46619 (1 × 10^−6^ M; Bio‐Techne, UK), ET‐1 (1 × 10^−8^ M; Bio‐Techne, UK), Ang‐II (1 × 10^−8^ M; Sigma, UK) or acetylcholine (1 × 10^−6^ M; Sigma, UK) or serial concentrations of histamine (1 × 10^−7^ to 1 × 10^−4^ M; Sigma, UK). These concentrations of agonists were selected based on previously published data in the relevant cell types. Images were taken at 0.2 s intervals for 2.5 min in one field of view. All images were taken on a Zeiss Axio Observer microscope (Carl Zeiss, Germany) using a 20×/0.8 DIC Plan Apochromat widefield objective and Hamamatsu Flash 4 camera. Cells were excited and emission filtered using a cyan (470/24) LED (Lumencor, USA, Spectra X) light source with a 470/40 nm, (Ex) 525/50 nm (Em) and 495 nm dichroic filter set. For analysis of intracellular Ca^2+^ dynamics ‘regions of interest’ were drawn around individual cells using Fiji software (v2.9; NIH, USA). All cells (approximately 100–400) that fulfilled the following inclusion criteria were selected: (i) only complete cells, (ii) only cells that remained attached to the base of the well throughout imaging and (iii) only cells that could be easily distinguished from other cells. Fluorescence was quantified using a bespoke ‘Intensity’ Fiji macro‐script following subtraction of background (F‐F_0_). The peak intensity (F‐F_0_) was determined using a customized macro and calculated across all cells.

### 2.7 2D Contraction Model and Data Analysis

ECFCs pre‐stained with Cell Tracker Red CMPTX (Thermo Fisher, UK) were plated (15,000 cells/well) onto 24‐well polymer glass‐like clear bottom black plates (Ibidi, Germany) in their regular media. The next day, the media was replaced with serum‐free (0% FBS) EGM2 and cells were incubated overnight at 37°C, 5% CO_2_ for cell quiescence. The next day, the media was replaced with ECS solution and cell contraction was imaged at ×20 magnification using a widefield Zeiss Axio Observer microscope, a Hamamatsu (Japan) Flash 4 camera, and a 562/40 nm and 624/40 excitation/emission filter set with a 593 nm beam splitter. Three random fields per well were selected before recording and images were taken at 2 min intervals for a total of 40 min. Cells were treated with either cumulative concentrations of histamine (1 × 10^−7^ to 1 × 10^−4^ M) or equivalent volumes of ECS (time control) from low to high concentrations at 10 min intervals. For analysis, all cells in each field were tracked individually using a bespoke macro‐script (https://www.imperial.ac.uk/medicine/facility‐for‐imaging‐by‐light‐microscopy/software/fiji/) for Fiji software (v2.9) which measured each cell's surface area overtime, normalized it to the cell size at the start of recording and expressed it as a percentage of control.

### 2.8 Statistical Analysis

Data are expressed as mean ± standard error of the mean (SEM) and analyzed using GraphPad Prism version 9. *N* values and statistical analyses used are defined in respective figure legends.

## 3 Results

### 3.1 ECFCs Derived From Donors With T1D Are Smaller but Grow With Similar Efficiency and Express Normal EC Markers Compared With Those Derived From Healthy Controls

**FIGURE 1:**
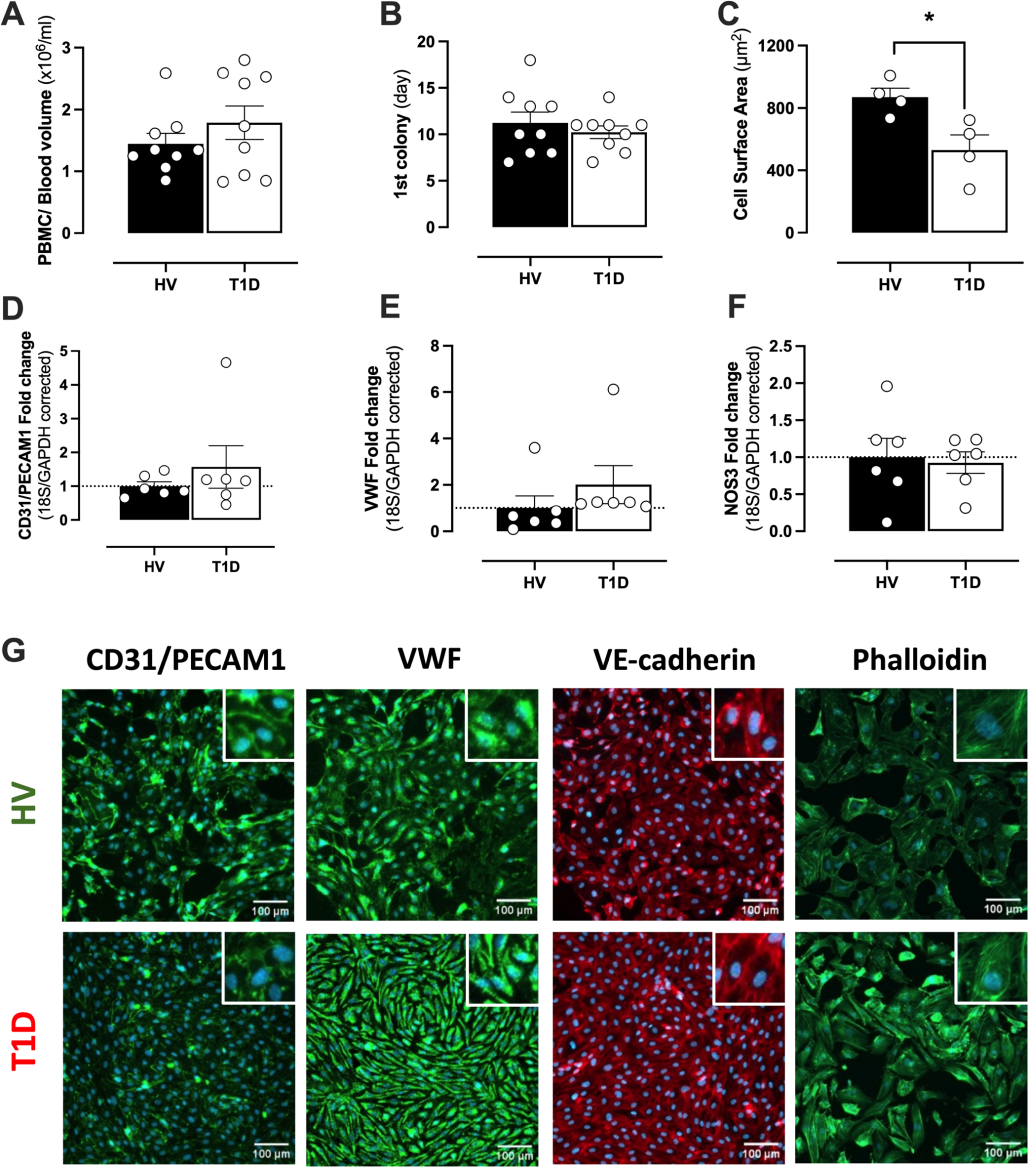
Characterization of ECFCs from control donors and donors with T1D. PBMC/blood count (A), day of first ECFC colony emergence (B) and total ECFC cell surface area (C). Gene expression of endothelial cells markers including CD31/PECAM1 (D), VWF (E), and NOS3 (F) were quantified using RT‐PCR. Cells were also stained antibodies against CD31/PECAM1, VWF, VE‐cadherin, and phalloidin (F‐actin) (G). Data are shown as mean ± SEM from *n* = 4–9 individual donors (control donors (HV); black bars and donors with T1D; white bars). For G, all cells were DAPI counterstained. Statistical difference was determined using an unpaired parametric *t*‐test. Significance was accepted where *p ≤ 0.05.

ECFCs are now routinely grown in numerous laboratories, yet the ability to isolate and grow these cells from human donors with T1D was unknown. We found no significant differences in PBMC counts from donors with T1D versus healthy controls (Figure 1A). The first appearance of outgrowth cells, after initial plating of PBMCs, occurred from Day 7 to 18. There was no difference in the average appearance of colonies between both groups (Figure 1B). ECFCs were initially identified by typical endothelial cell (cobblestone) and later characterized for various endothelial cell markers at gene/cell surface level. On average, cells from donors with T1D were significantly smaller than cells from control donors, although heterogeneity was seen between all donors (Figure 1C, Figure S1). Importantly, at the mRNA level ECFCs isolated from both groups expressed detectable levels (CT value< 35) of endothelial cell markers (CD31/PECAM1 (Figure 1D), VWF (Figure 1E) and NOS3 (Figure 1F)) (Figure 1D–F; Table S2). At the membrane level, ECFCs expressed high protein levels of endothelial cell markers CD31/PECAM1, VWF and VE‐Cadherin, and reacted to phalloidin (Figure 1G), but expressed negligible levels of the hemopoietic stem cell marker CD34 and smooth muscle cell marker ɑ‐SMA (Figure S2). There were no significant differences in the expression levels of phalloidin or any of the endothelial markers examined between T1D and healthy control ECFCs. There were also no significant differences in the expression levels of the proliferation markers PCNA, ENG and MK167 between T1D and healthy control ECFCs (Figure S3). It should be noted that not all colonies/cells grew to provide sufficient cell numbers from all donors for further analysis below.

### 3.2 ECFCs From T1D Donors Exhibit Exaggerated Expression of Cytokines and Adhesion Molecules Under Inflammatory Conditions

**FIGURE 2:**
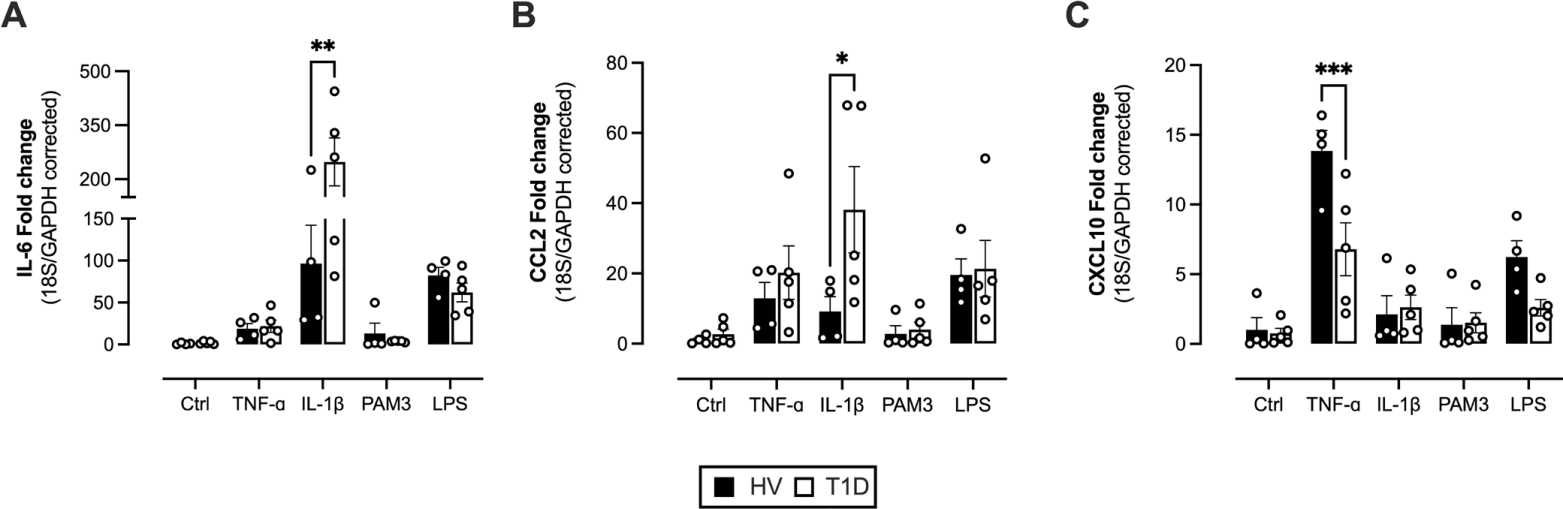
Effect of T1D on inflammatory marker (IL‐6, CXCL10, and CCL2) gene expression in response to inflammation‐inducing stimuli. ECFC gene expression of IL‐6 (A), CCL2 (B) and CXCL10 (C) after 24 h stimulation with either control or inflammatory stimuli (TNF‐α, IL‐1 β, PAM3 or LPS). Data are from *n* = 4–6 donors for control donors (black bars) or donors with T1D (white bars). Statistical difference was determined using a two‐way ANOVA with Sidak's post‐test. Significance was accepted where *p ≤ 0.05.

**FIGURE 3:**
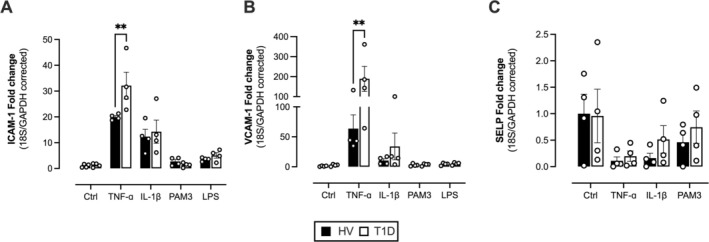
Effect of T1D on adhesion molecule (ICAM‐1, VCAM‐1 and SELP) gene expression in response to inflammation‐inducing stimuli. ECFC gene expression of the ICAM‐1 (A), VCAM‐1 (B) and SELP (C) after 24 h stimulation with either control or inflammatory stimuli (TNF‐α, IL‐1 β, PAM3 or LPS). Data are from *n* = 4–6 donors for control donors (black bars) and donors with T1D (white bars). Statistical difference was determined using a two‐way ANOVA with Sidak's post‐test. Significance was accepted where *p ≤ 0.05.

In response to inflammatory signals, endothelial cells upregulate important vascular mediators including adhesion molecules and pro‐inflammatory cytokines/chemokines and interferons, all of which are critical steps in diabetic vasculopathy. Induction of these mediators and adhesion molecules to regulate immune response is also triggered by type I cytokines IL‐1β and TNFɑ along with pathogen‐associated molecular patterns (PAMPs) for gram‐negative (LPS) and gram‐positive (Pam3CSK4) bacteria. We examined whether ECFCs derived from people with T1D were more sensitive to deleterious inflammatory signaling. Under basal/control culture conditions at 24 h ECFCs from both groups showed low and similar expression of the genes encoding IL‐6 (IL6), IP‐10 (CXCL10), and MCP‐1 (CCL2) (Figure 2A–C). After stimulation with inflammatory agents, mRNA expression for each cytokine/chemokine was elevated depending on the stimulus. T1D was associated with significantly greater induction of pro‐atherogenic/pro‐inflammatory cytokine/chemokine genes IL6 (Figure 2A) and CCL2 (Figure 2C) in IL‐1β‐treated cells compared to cells from healthy donors. In contrast, T1D was associated with reduced induction of the interferon marker, CXCL10, in response to TNFɑ (Figure 2B). It was not possible to validate these changes at the protein level, because the difference in cell size in these cultures (Figure 1C) would confound interpretation of mediator release data. The expression of the adhesion molecules ICAM‐1, VCAM‐1, and P‐selectin (SELP) under basal conditions was low and did not differ between groups (Figure 3A–C). ICAM‐1 (Figure 3A) and VCAM‐1 (Figure 3B) but not P‐selectin expression (Figure 3C) were induced strongly by TNFɑ and to a lesser extent by other stimuli. Induction of ICAM‐1 and VCAM‐1 in response to TNFɑ was significantly greater in cells from T1D donors compared to healthy controls (Figure 3A,B).

### 3.3 Histamine Elicits a Greater Rise in Intracellular Ca^2+^ Concentration in ECFCs Derived From Healthy Donors Compared to T1D Donors

**FIGURE 4:**
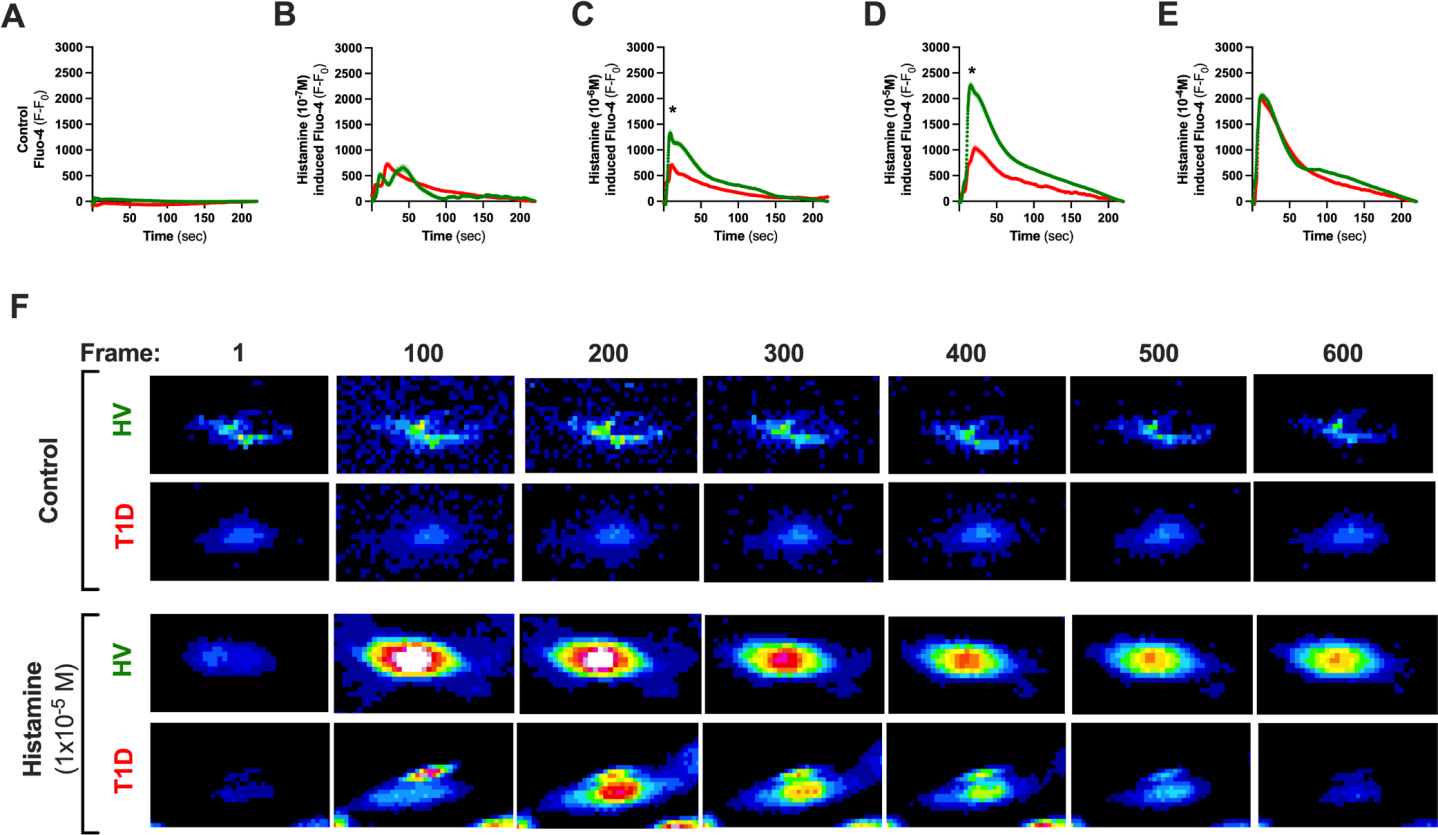
Effect of histamine on Ca^2+^ dynamics in ECFCs from controls and donors with T1D. Fluo‐4AM stained ECFCs from control donors (green) and donors with T1D (red) were treated with rising concentrations of histamine. To test the effect of histamine of Ca^2+^ dynamics in ECFCs, cells were treated in separate plates with control (ECS only) (A) and increasing concentrations of histamine including 1 × 10^−7^ M (B), 1 × 10^−6^ M (C), 1 × 10^−5^ M (D) and 1 × 10^−4^ M (E) for 5 min. Representative fluorescence images are shown (F). Data are from *n* = 4 control (HV) donors (green trace) and *n* = 3 donors with T1D (red trace). For A‐F, traces are from individual cells and include (A) *n* = 121 and *n* = 123, (B) *n* = 143 and *n* = 221, (C) *n* = 296 and *n* = 220, (D) *n* = 395 and *n* = 277, (E) *n* = 341 and *n* = 284 cells from control donors and donors with T1D respectively. Statistical difference was determined using a two‐way ANOVA with a Tukey's post hoc test. Significance was accepted where *p ≤ 0.05. Scale bar represents 50 μm.

**FIGURE 5:**
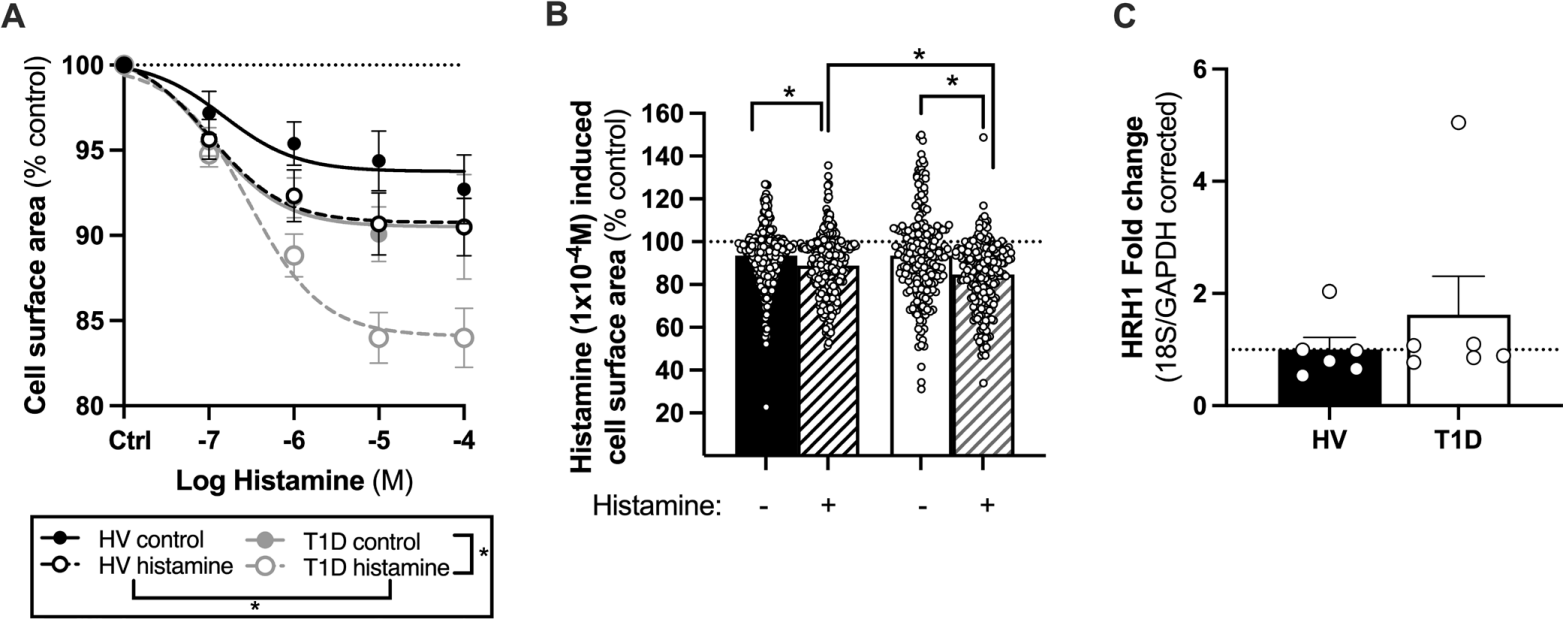
Effect of histamine on ECFC change in cell surface area and HRH1 expression in ECFCs. ECFCs isolated from control donors or donors with T1D were treated with control (ECS solution only) or cumulative concentrations of histamine (1 × 10^−7^–1 × 10^−4^ M) for 10 min per concentration. Contractile responses were measured as a change in cell surface area (%) from 3 randomly selected fields in each well (A). The effect of 1 × 10^−4^ M histamine for each individual cell was plotted (B). For (A) data are *n* = 12, where *n* = 4 individual donors (3 fields taken per donor) and for (B) data are shown as individual cells, where *n* = 259 and *n* = 323 cells from control donors or *n* = 212 and *n* = 246 cells from donors with T1D without and with histamine respectively. For (C), data are from *n* = 6 control donors and *n* = 6 donors with T1D. Statistical difference was determined using a two‐way ANOVA with a Tukey's post hoc test (A) or unpaired *t*‐test (B, C). Significance was accepted where *p ≤ 0.05.

Given the important role of histamine in diabetic vasculopathy, we sought to determine the effect of T1D on ECFC histaminergic Ca^2+^ responses. Measuring intracellular (IC) Ca^2+^ concentrations in ECFCs loaded with fluo‐4 showed a dose‐dependent rise in IC Ca^2+^ in response to histamine, which was significantly blunted at medium/high doses in ECFCs from T1D donors versus healthy volunteers (Figure 4) suggesting lower receptor sensitivity/potency to histamine in T1D. At the highest histamine concentration, similar maximum calcium responses were observed in both groups suggesting that all histamine receptors may have been blocked at this point or the assay may have reached the limit of detection. Next, we determined whether the effect of histamine on ECFC Ca^2+^ dynamics translated into cellular contraction (measured by the morphological change in cell surface area). Histamine caused a concentration‐dependent contraction in ECFCs from donors with T1D (Figure 5A). This trend was also true for ECFCs from control donors; however, the maximal effect of histamine at the highest concentration (*E*_max_) tested was significantly greater in ECFCs from donors with T1D. To elucidate whether these findings in ECFCs were due to differences in the expression of histamine receptors, we determined gene expression of HRH1, the most common histamine receptor in endothelial cells. Both ECFCs from control donors and donors with T1D expressed abundant levels of HRH1 (Table S2). No significant difference in the HRH1 expression levels was seen between both groups (Figure 5C).

## 4 Discussion

ECFCs are a proven and valuable research tool for a range of diseases, but little work has been carried out to characterize and test the functionality of ECFCs from people with diabetes, and ECFCs specifically phenotyped from donors with T1D have not been described. Here, we have exploited blood outgrowth cell technology to provide the first characterization of ECFCs grown from living donors with T1D and how they respond to inflammation and histamine, two key pathways involved in diabetic vasculopathy.

In our hands, PBMCs counts, ECFC day of colony emergence and isolation success rates (cells were isolated in > 70% of donors) were not altered by T1D. Other groups have reported that ECFCs grown from people with T2D show delayed colony emergence and a correlation between donor HbA1c and colony emergence [27, 28]. The reasons for such differences remain unknown but may be due to heterogeneity between donors, differences in culture protocols and/or differences in disease pathophysiology between T1D and T2D. Additionally, the influence of therapy on ECFCs colony formation and function must be considered. In the study of T2D ECFCs by Jarajapu et al. [28], most participants were not on statin therapy (48 out of 54 participants) while in our study most participants were on a statin. Statins have previously been shown to induce colony emergence, growth and survival in ECFCs isolated from pigs [29] and enhance the numbers of circulating endothelial progenitor cells in humans [30, 31]. Here we have shown that ECFCs can be effectively isolated from people with T1D. Further studies to unravel their utility as autologous sources of advanced cell therapies or to model T1D vasculopathy must ensure that all potentially confounding effects—from diabetes duration through to medications—are accounted for.

ECFCs are likely to originate from tissue vascular niches [32, 33]. Intravascular physical and chemical stimuli can influence endothelial cell morphology, which is crucial for establishing functional networks [34, 35]. It is reported that ECFCs grown from people with T2D have impaired cell motility/migration and reduced formation of microvasculature structure [19]. We show that ECFCs from donors with T1D are smaller in size in unstimulated conditions. The consequences of this observation on their regenerative potential, and how this could be rescued, pose an urgent question for future research.

Inflammatory activation of endothelial cells is a critical step in the development of diabetic vascular disease [36, 37]. We show that ECFCs from patients with T1D show exacerbated increases in mRNA levels of pro‐atherogenic/pro‐inflammatory genes (IL6 and CCL2) with IL‐1β treatment and reduced induction of the interferon marker (CXCL10) with TNFɑ compared to ECFCs from healthy donors. Human epidemiological studies have consistently found that IL‐6 is a risk marker for atherothrombotic events with genetic studies supporting a potential causal role for IL‐6 signaling in atherosclerosis [38]. Similarly, numerous mouse models have established CCL2, VCAM‐1 and ICAM‐1 as critical to increased lesion formation and recruitment of monocyte/macrophages in vessels [39, 40, 41, 42, 43]. Other published literature shows that coronary artery endothelial cells isolated from donors with T1D, when activated with inflammatory stimuli also have increased expression of IL‐6 and ICAM‐1 and increased NF‐kB activation [44] and that patients with T1D have elevated serum CCL2 [45], IL‐6 [5] and soluble VCAM‐1 [46], which correlate with disease complications including vascular disease. Only 2/9 of the donors with T1D in this study had retinopathy, hinting that the presence of established vascular complications is not the major driver of the altered phenotype observed in these cells. To what extent the differences are driven by ambient glucose, duration of elevated HbA1C or other factors remains to be discovered.

The suppressed induction of interferon signaling markers (IP‐10) seen in ECFCs from T1D donors is predictive of an abnormal response to viral infection. In alignment with this, two population‐based analyses from the UK have clearly shown a higher mortality in patients with T1DM compared with a population without T1DM supporting the association between T1DM and poor COVID‐19 outcomes [47, 48]. The mechanisms for these modulated inflammatory responses are unclear but these cells will now provide a unique platform with which to explore this and how it could be therapeutically targeted in patients with T1D.

Histamine disrupts vascular endothelial barrier function during inflammation, a feature of vasculopathy and even the development of T1D islet destruction itself [49, 50]. Here we show that ECFCs from donors with T1D show impaired intracellular Ca^2+^ signaling and hypercontractile responses to histamine without differences in histamine receptor expression. These observations illustrate the complex nature of intracellular Ca^2+^ dynamics and contraction and it will be interesting to see future studies that validate these findings using other assays of barrier function. The divergence between intracellular Ca^2+^ and contractile responses in ECFCs from T1D may reflect a heightened sensitivity to Ca^2+^ signaling within the cell. This idea remains the subject of investigation. In agreement with our findings, cardiac endothelial cells isolated from diabetic rats have been shown to have a smaller Ca^2+^ peak amplitude compared to wild‐type rats when treated with contractile agonists [51]. In humans, plasma histamine concentrations are elevated in diabetes and peripheral vascular disease [24] and endothelial cells may therefore be primed. It will be interesting to investigate whether any of these histaminergic aberrations can be reprogrammed in diabetic ECFCs—both in order to improve their utility for cell therapy and to identify new targets to treat diabetic vasculopathy.

In summary, we have characterized for the first time the basic morphology of ECFCs from donors living with T1D, investigated their response to inflammatory stimulation and measured the effects of histamine on Ca^2+^ signaling and contraction. This pilot work shows distinct phenotypic differences in ECFCs from T1D donors compared with cells derived from healthy volunteers, and preserved phenotypes that indicate their usefulness for studying endothelial inflammation and dysfunction in T1D. ECFCs clearly provide a useful model for mechanistic studies, personalized medicine, drug screening and tissue engineering in diabetes and other vascular diseases. Their application in T1D is now established and likely to be an area of intensive future research.

## Author Contributions

Blerina Ahmetaj‐Shala, Nicholas Oliver, and Jane A. Mitchell conceived and designed the research. Isra Marei, Maria Vinokurova, Li Qiucheng, Lasse Nyhegn, Anam Baig, and Nura Mohamed performed the research and acquired and analyzed the data. Eric Dubuis, Stephen Rothery, and Nicholas S. Kirkby assisted with data interpretation and analysis. Blerina Ahmetaj‐Shala, Jane A. Mitchell, and Victoria Salem wrote the manuscript. All authors were involved in checking and revising the manuscript.

## Conflicts of Interest

The authors declare no conflicts of interest.

## Supporting information


**Data S1:** fba270066‐sup‐0001‐DataS1.pdf.

## Data Availability

The data that support the findings of this study are available on request from the corresponding author.
