# Supplementary material for: Impact of Type 1 Diabetes on Endothelial Cells Derived From Living Donors
Source: FASEB Bioadv. 2025 Nov 5;7(11):e70066. doi: 10.1096/fba.2025-00104 (PMC12587046; doi:10.1096/fba.2025-00104)
Supplement: Supplementary file 1 — Data S1: fba270066‐sup‐0001‐DataS1.pdf. [file FBA2-7-e70066-s001.pdf]

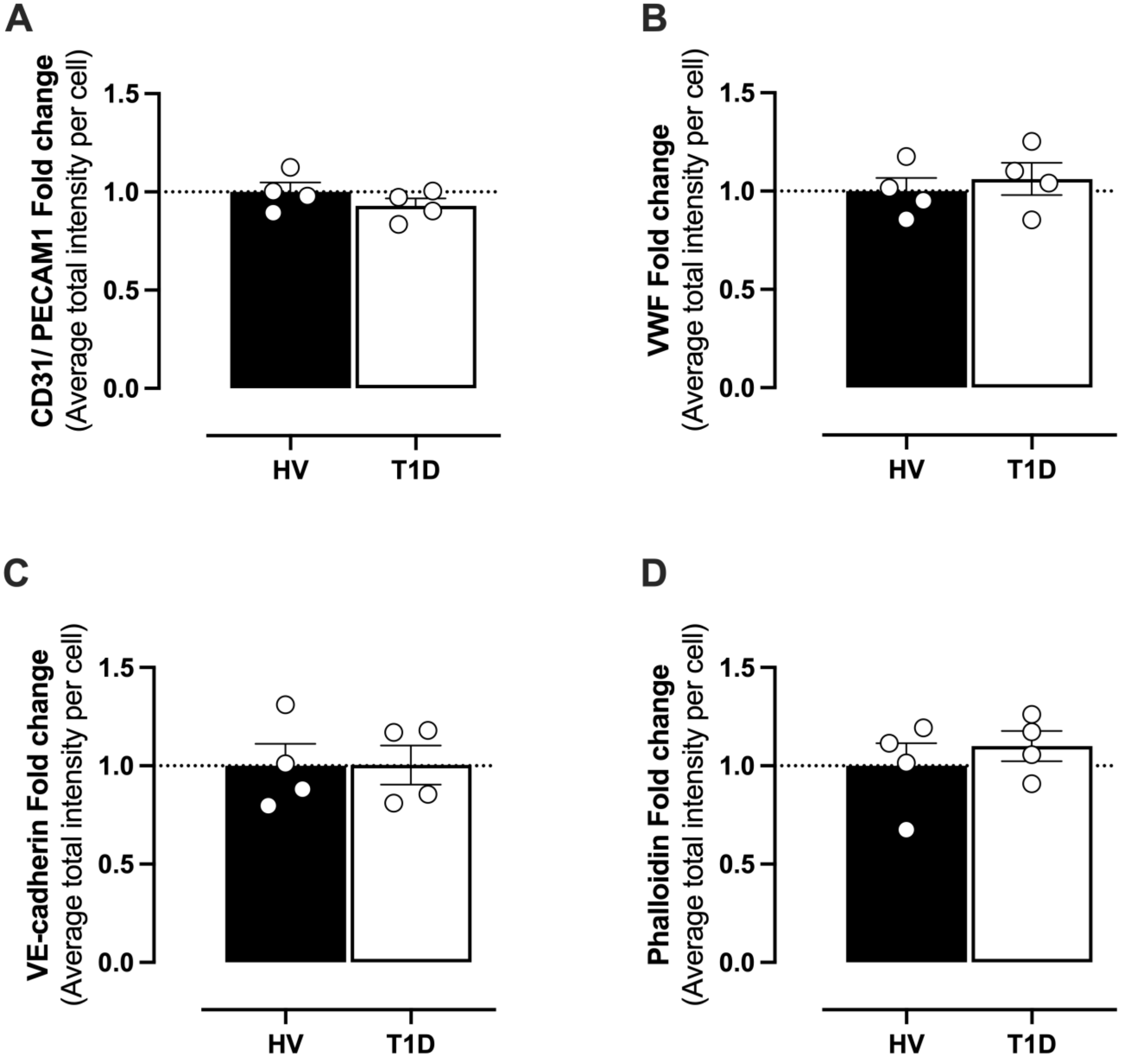

Figure S1: Average total intensity per cell of ECFCs stained with endothelial cell markers. ECFCs were stained with CD31/PECAM1 (A), VWF (B), CE-cadherin (C) and phalloidin (D) and imaged. Three separate fields were selected per donor from n=1-2 separate experiments. Using a plugin/macro for FIJI, the intensity was subtracted from the background and divided by the number of cells (determined by DAPI staining). All fields were averaged to yield one value per donor and divided by the average of the control donors. Data are from ECFCs from n=4 control donors and n=4 donors with T1D. Statistical difference was determined using an unpaired t-test and accepted where $p<0.05$.

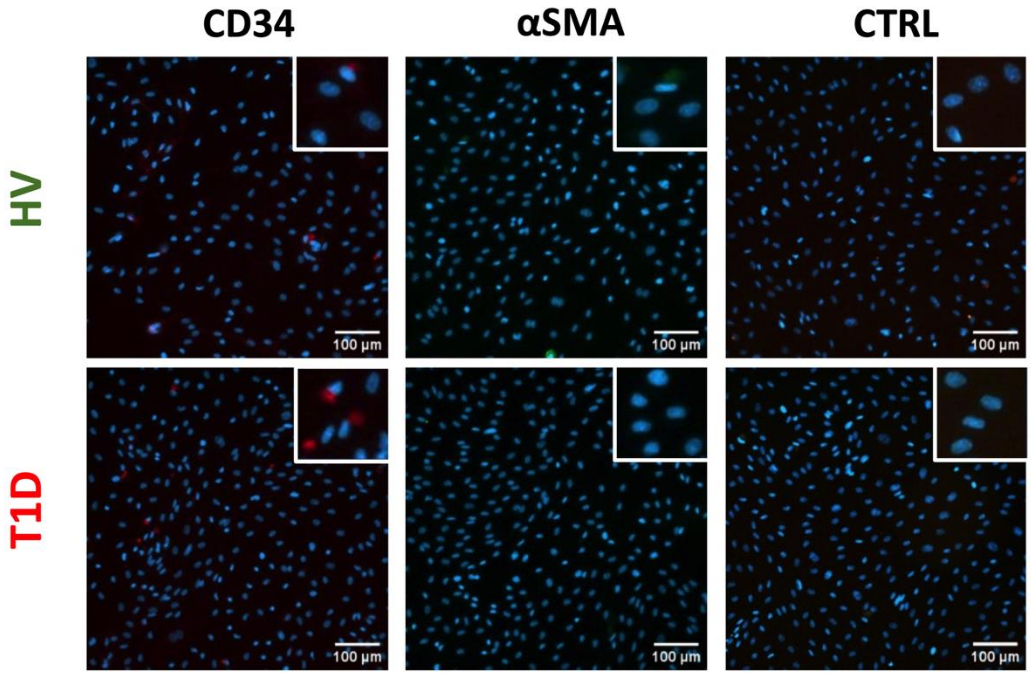

Figure S2: Characterisation of ECFCs using immunohistochemistry. As a control, ECFCs were stained with the hematopoietic progenitor stem cell marker CD34 (red), the smooth muscle cell marker a-SMA (green) and control (DAPI only; blue). Three random fields were chosen for imaging per well. Representative images shown are from n=1-2 experiments from n=4 control donors and n=4 donors with T1D.

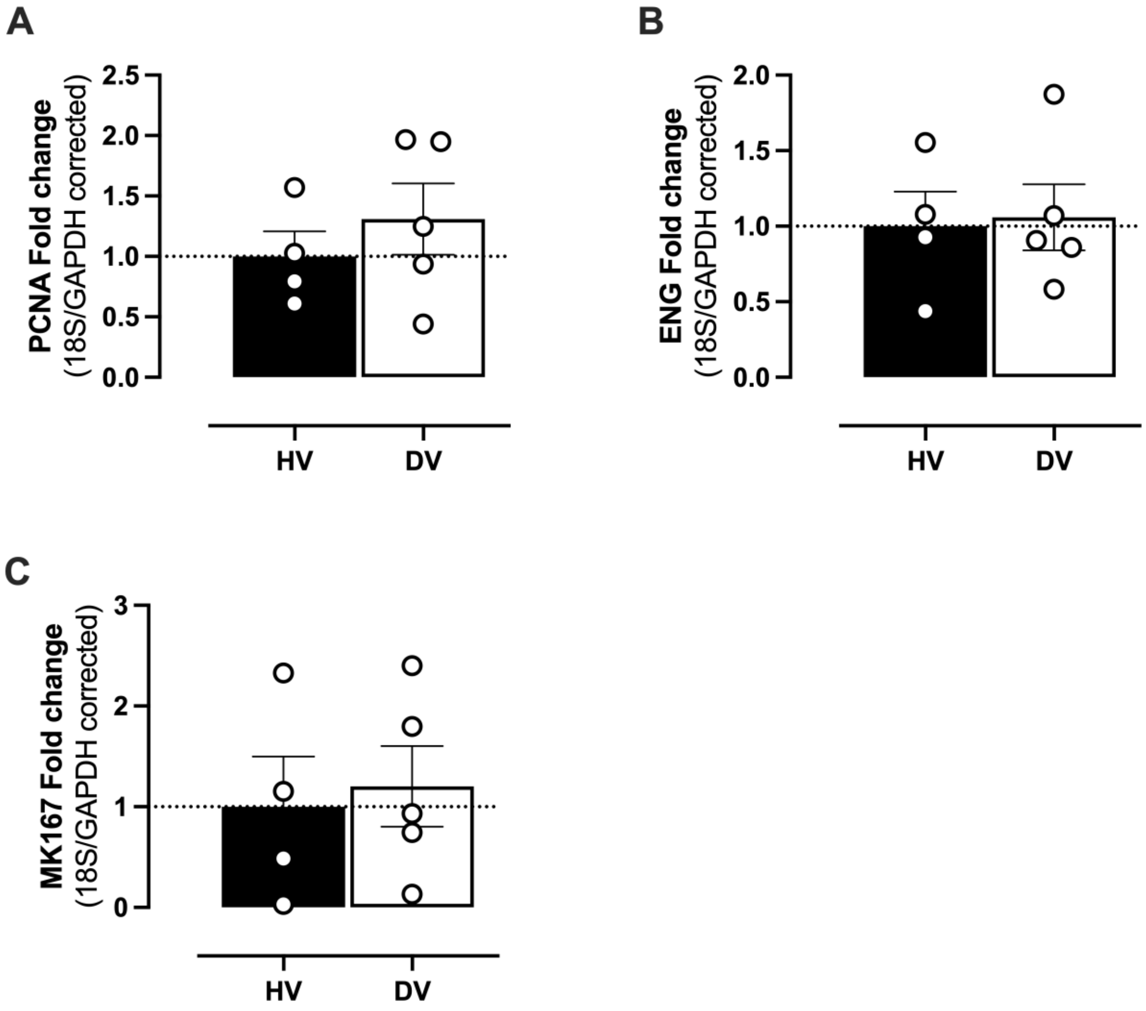

Figure S3: Effect of T1D on proliferation marker (PCNA, ENG and MK167) gene expression. ECFC gene expression of PCNA (A), ENG (B) and MK167 (C) after 24 hours in basal condition. Data are from n=4-6 donors for control donors (black bars) or donors with T1D (white bars). Statistical difference was determined using a two-way ANOVA with Sidak's post-test. Significance was accepted where *$p \leq 0.05$.

Antibody / dye	Company	Dilution
**Alexa FluorTM 488-Conjugated Anti-Human CD31**	BioLegend	1:100
**Polyclonal Rabbit Anti-Human Von Willebrand Factor (VWF)**	Dako	1:200
**Polyclonal Goat Anti-Human VE- Cadherin**	Santa Cruz Biotechnology	1:100
**PerCP-CyTM5.5-Conjugated Monoclonal Mouse Anti-Human CD34**	BD Bioscineces	1:100
**Alexa FluorTM 488-Conjugated Alpha-Smooth Muscle Actin (αSMA) Monoclonal Antibody**	Invitrogen	1:500
**Alexa FluorTM 488-Conjugated Goat Anti-Rabbit**	Invitrogen	1:2000
**Alexa FluorTM 594-Conjugated Donkey Anti-Goat**	Invitrogen	1:2000
**FITC-Conjugated Phalloidin**	Abcam	1:1000
**DAPI**	Invitrogen	1:2000

Table S1: Information of antibodies and dyes used in immunocytochemistry staining of ECFCs. Primary antibodies include antibodies conjugated with Alexa Fluor (AF)TM 488/ PerCP- CyTM5.5, and unconjugated antibodies which require the binding of secondary antibodies with conjugated fluorescent probes.

Cell type	HV ECFCs (CT values)					
**Gene**	Donor 1	Donor 2	Donor 3	Donor 4	Donor 5	Donor 6
**18S/ GAPDH average**	16.2	14.6	16.7	15.2	17.2	17.2
**CD31/PECAM1**	18.9	17.2	18.6	18.3	19.2	20.0
**VWF**	20.0	18.0	17.7	18.6	20.8	23.3
**NOS3**	30.0	29.0	31.4	30.1	29.9	34.5
**ICAM1**	31.8	29.3	32.0	30.8	31.6	30.7
**IL-1ß**	32.4	32.7	32.5	32.5	34.3	32.8
**IL-6**	26.4	26.6	28.0	27.6	28.2	28.0
**IL-8**	19.9	20.9	22.4	21.1	22.4	20.1
**HRH1**	26.0	24.5	26.1	25.5	26.5	25.5
**Cell type**	**T1D ECFCs (CT values)**					
**Gene**	Donor 1	Donor 2	Donor 3	Donor 4	Donor 5	
**18S/ GAPDH average**	14.9	16.4	15.2	19.7	16.4	
**CD31/PECAM1**	17.8	18.6	18.8	19.9	18.7	
**VWF**	17.1	18.1	17.6	18.6	17.7	
**NOS3**	28.5	29.9	30.9	32.7	30.1	
**ICAM1**	30.3	31.2	30.2	33.0	31.5	
**IL-1ß**	33.5	33.9	34.9	Undetected	Undetected	
**IL-6**	26.6	27.3	26.0	29.4	27.8	
**IL-8**	21.6	22.6	22.2	24.4	23.2	
**HRH1**	24.7	26.0	24.5	26.7	25.6	

Table S2: Raw cycle threshold (CT) values detected using RT-PCR of genes of interest of ECFCs isolated from control (HV) donors and donors with type 1 diabetic (T1D). Raw CT values below 35 were considered to be undetected. Experiments were conducted using samples of ECFCs isolated from n=6 HV and n=5 T1D donors.
